# Knowledge of obstetric danger signs and associated factors among reproductive age women in Raya Kobo district of Ethiopia: A community based cross-sectional study

**DOI:** 10.1186/s12884-017-1253-4

**Published:** 2017-02-21

**Authors:** Nigus Bililign, Tesfahun Mulatu

**Affiliations:** 1 Department of Midwifery, Faculty of Health sciences, Woldia University, PO.box: 400 Woldia, Ethiopia; 2 Department of Public Health, Faculty of Health sciences, Woldia University, Woldia, Ethiopia

**Keywords:** Obstetric danger sign, Pregnancy, Delivery, Postpartum, Ethiopia

## Abstract

**Background:**

Knowledge of danger signs of obstetric complications during pregnancy, labour, and postnatal period is the first essential step for appropriate and timely referral. This study aimed to investigate predictors of knowledge about obstetric danger signs during pregnancy, delivery, and postpartum period among mothers of reproductive age women who gave birth in the last 12 months.

**Methods:**

A quantitative community based cross-sectional study was employed during March 2016. The study included 493 mothers who were selected by multi-stage sampling technique. Data were collected by face-to-face interview. Logistic regression analyses were employed to identify factors associated with knowledge of obstetric danger signs. Variables with a *p*-value <0.05 were identified as statistically significant factors.

**Results:**

About forty-seven percent (46.7%), 27.8%, and 26.4% of the mothers were knowledgeable about obstetric danger signs during pregnancy, delivery, and postpartum period, respectively. Vaginal bleeding was the most frequently cited danger sign during the three periods. Mothers’ secondary or above education level increased odds of knowledge about danger signs during pregnancy (AOR: 3.63; 95% CI 1.19, 11.07) and postpartum period (AOR: 5.31; 95% CI 2.13, 13.22). Additionally, being employed (AOR: 5.41; 95% CI 1.03, 28.32), delivery at health institution (AOR: 2.14; 95% CI 1.17, 3.92) and number of ANC visits were statistically significant factors.

**Conclusion:**

Knowledge of mothers about obstetric danger signs was low in the study area. Empowering women, improving the quality of health information about danger signs during ANC follow up, and promoting institutional delivery are the recommended interventions.

## Background

As the 2015 target date for the Millennium Development Goals (MDGs) neared, preventable causes of maternal mortality remained the world’s most critical challenges despite significant progress over the past decade. Although the global maternal deaths have decreased by 45% since 1990, 800 women still die each day from largely preventable causes before, during, and after the time of giving birth [1]. In 2015, about 99% (302,000) of the global maternal deaths were in developing regions, with sub-Saharan Africa accounting for 66% (201,000) [2]. For every 100,000 live births, 450 women died during the three periods (pregnancy, childbirth, or postpartum) in developing countries [3]. The United Nations’ (UN) Sustainable Development Goals (SDGs) has set a new transformative agenda for maternal health to reduce the global maternal mortality ratio (MMR) to less than 70 per 100 000 live births by 2030 (SDG 3.1) [4]. Ethiopia has made notable progress in decreasing maternal mortality ratio from 676 in 2011 [5] to 353 in 2015 [2] per 100,000 live births.

Delay in seeking care is one of the key factors leading to maternal death, which can be associated with lack of knowledge about obstetric danger signs [6]. Severe vaginal bleeding, swollen hands/face and blurred vision are the key danger signs during pregnancy. The key danger signs during labor and childbirth include: severe vaginal bleeding, prolonged labor (>12 h), convulsions and retained placenta. Additionally, severe vaginal bleeding, foul-smelling vaginal discharge and high fever are the key danger signs during the postpartum period [7].

In Ethiopia, hemorrhage, hypertension in pregnancy, abortion and sepsis are the leading causes of maternal deaths, which can be averted through recognition of danger signs of these complications and skilled institutional care. The Ethiopian federal ministry of health plans to reduce the MMR to 199/100,000 live births by 2020 [8].

A community-based cross-sectional study conducted in Jimma zone showed that the study participants mentioned severe vaginal bleeding (26.7%), swollen hands/feet (14.7%) and blurred vision (29.8%). Additionally, severe vaginal bleeding (49.5%), convulsion (16.3%), prolonged labor (14.9%) and retained placenta (15.1%) were recognized as danger signs during labour and delivery. During the postpartum period vaginal bleeding (45.3%), foul smelling vaginal bleeding (15.2%) and high fever were key danger signs mentioned by the study participants (12.1%) [9]. A qualitative study in Kenya showed heavy vaginal bleeding before expected date of delivery, unpleasant vaginal discharge, water breaking before due date, abnormal presentations abdominal pain and dizziness were cited as obstetric danger signs by the respondents [10].

A cross-sectional study conducted in rural Tanzania revealed maternal education level, number of antenatal care (ANC) follow-ups and place of delivery were predictors of mothers’ awareness about obstetric danger signs during pregnancy, delivery and postpartum period [11]. Another study conducted in Goba district of Ethiopia showed that, 31.9%, 27% and 22.1% of the study participants were knowledgeable about danger signs during pregnancy, delivery, and postpartum period respectively. Place of residence, mother and husband educational status, mothers’ occupation, and ANC follow-up were found to be significant factors for knowledge of danger signs [12].

Other studies in East Gojjam and Tsegedie district of Northern Ethiopia showed institutional delivery were associated with increased odds of knowledge about danger signs during pregnancy, delivery, and postpartum period [13, 14].

Awareness of obstetric danger signs during pregnancy can increase women’s capacity to ensure timely skilled care and safe birth [15]. Knowledge of obstetric danger signs during the three periods is an important first step for appropriate management and timely referral. It is also a better strategy to enhance skilled and emergency obstetric cares in low-income countries [3]. Increasing knowledge of obstetric danger signs for pregnant women would reduce delay in seeking care and improve early detection of obstetric complications [3, 6]. This study aimed to investigate knowledge of obstetric danger signs and associated factors among women of reproductive age in Raya Kobo district, North-eastern Ethiopia.

## Methods

### Study area and period

The study was carried out in Raya Kobo district, North Wello zone, Ethiopia during March 2016. Raya Kobo district is one of the thirteen districts of North Wello Zone, which is located 570 km northeast of Addis Ababa. This district has an estimated total population of 228,798. The number of reproductive age women (15–49) and pregnant women in the district is 53,951 and 7,710, respectively. There are forty-two kebeles (the smallest administrative units) in Raya Kobo district: five urban kebeles and thirty-seven rural kebeles. In this district, there are forty-two health posts, seven health centers and eight private clinics with two health extension workers in each health post. [*North Wello health sector annual report, 2015: Unpublished].*

### Study design and population

A community-based cross-sectional study design was employed. mothers who had been living for at least six months in the district were the source population of this study and who had given birth within the last 12 months prior to the data collection, regardless of their birth outcome.

### Sample size determination

The sample size was determined using a formula for single population proportion as follows: proportion of mothers knowing obstetric danger signs during pregnancy to be 30.9% [12], confidence level 95%, absolute precision ±5%, design effect 1.5, and non-response rate 5%. Thus, the final sample size was 517.

### Sampling procedure

Stratification was done into urban (*n* = 5) and rural (*n* = 37) kebeles. In each stratum, 1/3 of the kebeles (two urban and 12 rural) kebeles were selected by simple random sampling method. Census was done in the selected fourteen kebeles to identify mothers who gave birth in the last one year prior to the data collection. Then, the total sample size (*n* = 517) was allocated proportionally (443 and 74 mothers from rural and urban kebeles respectively) on each kebele based on the number of eligible mothers present in the area. Finally, study subjects in each kebele were selected using systematic sampling method. Lottery method was employed to select the first household in each kebele.

### Operational definitions

#### Knowledge of obstetric complication(s)

Any symptom of obstetric complications mentioned by the study participant, which occur in women during the three periods (pregnancy, delivery, or postpartum).

#### Knowledgeable on key danger signs of pregnancy, delivery and postpartum period

In this study a woman is considered as knowledgeable about danger signs of obstetric complications if she can mention at least three key obstetric danger signs for each of the three periods (pregnancy, delivery and postpartum) spontaneously or after prompting. Prompting was done by providing the mothers with more time in helping them to try and remember these periods.

### Data collection procedure and quality control

Data were collected using structured questionnaire adapted from the Maternal and Neonatal Program of JHPIEGO, an affiliate of John Hopkins University [3]. The questionnaire was first translated from English to local language (Amharic) and back to English. Pre-test was done on 5% of the total sample size in Woldia town and a necessary adjustment was made. Eight diploma Midwives who are fluent in speaking local language were involved in the data collection. Two Bachelor of Science degree holder health professionals were recruited as supervisors.

Internal consistency (reliability) was tested by calculating Cronbach’s Alpha using statistical package for social sciences (SPSS) window version 20. Furthermore, maternal health expert crosschecked content validity of the tool. Data collectors and supervisors were trained about the study instrument and data collection procedure. The principal investigator and the supervisors checked the collected data for completeness.

### Data processing and statistical analysis

SPSS computer software was used to code and enter the data. After checking its completeness and consistency of the tool, further clearance was made using this software. The level of knowledge on key danger signs of obstetric complications during the three periods and other independent variables like socio-demographic and obstetric variables were described. Additionally, binary logistic regression analysis was employed to see the single effect of an independent variable during pregnancy, delivery and postpartum period. Furthermore, multiple logistic regression analyses were employed to identify existence of relationship between the outcome variables (knowledge of danger signs during pregnancy, delivery and postpartum period) and selected associated factors. Candidate variables for multiple logistic regression analysis, variables with a *p*-value less than 0.05 in the univariate analysis, were entered into a multiple logistic regression analysis. On the top of this, the effect of confounding was controlled. Variables with a *P*-value <0.05 were considered as independent predictors of knowledge about obstetric danger signs.

### Ethical approval

Institutional Research Review Board (IRRB) of Woldia University approved the proposal of this research. Letter of permission was obtained from North Wello Zone health sector, Raya Kobo district administrative and health offices. Informed verbal consent was obtained from the study subjects after the data collectors explained the study objectives, procedures and their right to refuse not to participate in the study. Furthermore, confidentiality of the study subjects was assured.

## Results

### Socio demographic characteristics of participants

A total of 493 mothers were included in the study, yielding a response rate of 95%. The mean age of the study participants was 29.0 (SD ± 7.56). Ethiopian Orthodox Christianity (67.7%) was the dominant religion and 72.2% of the respondents were married women. About half (49.1%) of the respondents were illiterate and housewives account 88.4% [Table 1].

**Table 1:** Socio demographic characteristics of respondents in Raya Kobo district, North-eastern Ethiopia, March 2016

Variables (*n* = 493)	Frequency	Percent
Age <=20 21–25 26–30 >30	50 135 133 175	10.1 27.4 27.0 35.5
Religion Orthodox Muslim Others*	334 158 1	67.7 32 0.2
Ethnicity Amhara Others**	491 2	99.6 0.4
Marital status Married Single Widowed Divorced Separated	356 10 20 76 31	72.2 2.0 4.1 15.4 6.3
Mothers’ education illitrate read and write only primary secondary and above	242 56 160 35	49.1 11.4 32.5 7.1
Mothers’ occupation house wife gov’t employee private merchant others***	436 8 15 22 12	88.4 1.6 3.0 4.5 2.4
House hold income (ETB) <=100 101–300 > = 301	293 75 125	59.4 15.2 25.4

*protestant; **tigre; ***daily laborer, student; ETB-Ethiopian birr

### Obstetrical characteristics

Forty percent of the respondents spent more than thirty minutes to reach health institutions from their home. 72.6% of the study participants had at least one antenatal care (ANC) follow-up for their last pregnancy. Majority of the respondents start their ANC visit at or less than 16 weeks of gestational age. Many mothers (43.4%) gave birth to their last child at home [Table 2].

**Table 2:** Obstetrical characteristics of respondents in Raya Kobo district, North-eastern Ethiopia, March 2016

Variables (*n* = 493)	Frequency	Percent
Time spent to health institution <15 min 15–30 min >30 min	217 79 197	44 16 40
Gravidity 1 2–4 > = 5	107 273 113	21.7 55.4 22.9
Antenatal care visit (ANC)* Yes No	358 135	72.6 27.4
Frequency of ANC visit 1 2–4 > 4	10 197 151	2.5 55 42.2
Gestational age during first ANC visit (weeks) <=16 17–24 25–32 > = 33	248 73 32 2	69.3 20.4 9.8 0.6
Place of delivery Home Health institution	214 279	43.4 56.6

*at least one visit

### Knowledge of obstetric danger signs

More than half (53.3%) of mothers were not knowledgeable (knew fewer than three obstetric danger signs) about danger signs during pregnancy.

On the other hand, 356 (72.2%) of the mothers were not knowledgeable about obstetric danger signs during labour. Similarly, 363 (73.6%) of the study participants were found to be not knowledgeable about obstetric danger signs during postpartum period.

Vaginal bleeding (83.5%) and accelerated/decreased fetal movement (38.1%) were the most frequently mentioned obstetric danger signs during pregnancy by the study participants. Additionally, vaginal bleeding (91.2%) and retained placenta (58.7%) were the most frequently mentioned complications during labour. During the postpartum period, vaginal bleeding (89.2%), offensive vaginal discharge (23.3%) and severe headache (23.1%) were the most frequently known complications by mothers in the study [Table 3].

**Table 3:** Knowledge of mothers about obstetric danger signs during pregnancy, delivery and postpartum period in Raya Kobo district, North-eastern Ethiopia, March 2016

Danger signs	Knowledge of danger signs during					
	Pregnancy		Labour & delivery		Postpartum	
	N	%	N	%	N	%
Vaginal bleeding	364	83.5	415	91.2	371	89.2
Severe headache	107	24.5	46	10.1	96	23.1
Blurred vision	34	7.8	NA	NA	37	8.9
Convulsion	25	5.7	12	2.6	33	7.9
Swollen hands/feet	56	12.8	NA	NA	88	21.2
High fever	23	5.3	15	3.3	21	5
Loss of consciousness	35	8	36	7.9	35	8.4
Difficulty in breathing	15	3.4	NA	NA	15	3.6
Severe weakness	71	16.3	NA	NA	77	18.5
Severe abdominal pain	130	29.8	NA	NA	NA	NA
Increased/decreased fetal movement	166	38.1	NA	NA	NA	NA
Water breaks without labor	155	35.6	NA	NA	NA	NA
Labor > 12 h	NA	NA	129	28.4	NA	NA
Retained placenta	NA	NA	267	58.7	NA	NA
Offensive vaginal discharge	NA	NA	NA	NA	97	23.3
Others	6	1.4*	48	10.5**	12	2.9***

NA = not assessed for that period, *abnormal lie, **abnormal presentation, ***abdominal cramping

### Factors associated with knowledge of obstetric danger signs

Mothers’ education was found to be significantly associated with knowledge of key danger signs during pregnancy and postpartum period. Mothers who attend secondary education were more likely to be knowledgeable about danger signs during pregnancy than their illiterate counterparts (AOR: 3.63; 95% CI 1.19, 11.07). Similarly, women who attend secondary education were about five times more likely to know danger signs of postpartum period than illiterate mothers (AOR: 5.31; 95% CI 2.13, 13.22). Mother’s occupation was another significant factor for knowledge of obstetric danger signs during pregnancy. Private employees were more knowledgeable about pregnancy danger signs than housewives (AOR: 5.41; 95% CI 1.03, 28.32). However, it did not show significant association with knowledge of danger signs during labour and postpartum period.

The number of antenatal care visits was another variable found to be significantly associated with knowledge of mothers about danger signs during pregnancy. Mothers’ who visit ANC clinic > =4 times were 11 times more likely to be knowledgeable about danger signs during pregnancy than mothers’ who had only one visit (AOR: 0.09; 95% CI 0.01, 0.81). Mothers’ who gave birth to their last child at health institutions were about two times more knowledgeable about labour danger signs than their counterparts who gave birth at home (AOR: 2.41; 95% CI 1.17, 3.92) [Table 4].

**Table 4:** Factors associated with knowledge of obstetric danger signs during pregnancy, labour and postpartum period in Raya Kobo district, North-eastern Ethiopia, March 2016

Variables	AOR (95% CI) for knowledge about danger signs of pregnancy	AOR (95% CI) for knowledge about danger signs of labour	AOR (95% CI) for knowledge about danger signs of postpartum
Mothers’ education Illiterate Read & write only Primary Secondary & above	1 0.69 (0.29, 1.60) 1.45 (0.83, 2.54) **3.63** (**1.19**, 11.07)*	1 0.70 (0.31, 1.57) 0.96 (0.56, 1.65) 1.79 (0.74, 4.33)	1 1.15 (0.50, 2.64) 1.27 (0.72, 2.23) **5.31**(**2.13**, 13.22)*
Husband education Illiterate Read & write only Primary Secondary & above	1 1.74 (0.94, 3.21) 1.08 (0.52, 2.24) 0.72 (0.29, 1.82)	**	1 1.46 (0.80, 2.69) 1.11 (0.53, 2.32) 1.51 (0.64, 3.55)
Household income <=100 101–300 > = 300	1.09 (0.57, 2.08) 0.87 (0.38, 2.00) 1	**	**
Mothers’ occupation Housewife Private employee Merchant Other	1 **5.41**(**1.03**, 28.32)* 1.81 (0.48, 6.82) 2.26 (0.16, 30.42)	1 2.30 (0.76, 6.97) 1.50 (0.53, 4.26) 1.95 (0.43, 8.83)	1 2.16 (0.60, 7.84) 1.41 (0.43, 4.63) 0.541 (0.04, 6.35)
Number of ANC visit 1 2–3 > = 4	**0.09** (**0.01**, 0.81)* **0.53** (**0.31**, 0.90)* 1	1.55 (0.33, 7.16) 0.59 (0.35, 0.99) 1	**
Place of delivery Home Health institution	1 0.808 (0.453, 1.441)	1 **2.14** (**1.17**, 3.92)*	1 0.97 (0.57, 1.67)
Distance from nearest HF <15 min 15–30 min > = 30 min	**	1 1.64 (0.89, 3.02) 0.76 (0.41, 1.39)	1 1.32 (0.67, 2.56) 0.78 (0.44, 1.38)

AOR adjusted odds ratio, CI confidence interval, HF health facility, *statistically significant variables at *P*-value <0.05 (adjusted for all variables in the table), **P-value of the variable was greater than 0.05 which was not a candidate for logistic regression

## Discussion

This study showed that 46.7%, 27.8% and 26.4% of the study participants were knowledgeable about key danger signs of pregnancy, delivery and postpartum period, respectively. This finding is higher than studies done in rural Tanzania and Somali regional state of Ethiopia [11, 16]. This difference might be due to relatively high antenatal care visit coverage in this study. The finding of this study is consistent with a study conducted in Goba district during labour and delivery (27%) [12]. However, higher prevalence of knowledge about danger signs was reported during postpartum period and delivery [17, 18]. Knowledge of mothers in the three periods is lower than findings of similar cross-sectional study conducted in Wolayita zone of Ethiopia [19]. A mother was considered as knowledgeable if she could mention two danger signs but in the current study, the mother had to mention at least three danger signs to be considered knowledgeable.

This study showed that, vaginal bleeding was the most frequently mentioned danger sign during pregnancy (83.5%), delivery (91.2%), and postpartum period (89.2%). This finding is consistent with other studies conducted in different parts of Ethiopia and other countries [9, 12, 17, 18, 20]. Accelerated/decreased fetal movement (38.1%) and water breaks without labor (35.6%) were other frequently mentioned danger signs during pregnancy by mothers in this study. Lower prevalence of knowledge about these danger signs were reported by a study in Tsegedie district of Ethiopia [14]. In this study, retained placenta and prolonged labour were known by 58.7% and 28.4% of the study participants during delivery, respectively. This finding is in line with a study conducted in Goba district of Ethiopia [12]. Additionally, offensive vaginal discharge (23.3%) and severe headache (23.1%) were postnatal danger signs mentioned by the respondents of this study. Similarly, in studies in Kenya and Ethiopia offensive vaginal discharge was frequently mentioned as a postpartum danger sign [10]. However, high fever was mentioned by only 5% of the study participants of this research. This figure is lower than findings from Goba and Tsegedie districts of Ethiopia [12, 14].

In this study, maternal educational status was significantly associated with knowledge about obstetric danger signs during pregnancy and postpartum period. Mothers having secondary or higher education level were 3.6 and about five times more likely to be knowledgeable about pregnancy and postpartum danger signs respectively than illiterate mothers. Similar finding was reported by a study conducted in rural Tanzania where mothers who attend secondary or higher education were about six times more likely to recognize obstetric danger signs [11]. Another study conducted in Nigeria showed ever attending education was associated with increased odds of knowing obstetric danger signs [19]. Similar study conducted in East Gojjam zone of Ethiopia showed maternal educational status (secondary school) increased odds of knowledge about obstetric danger signs by two fold [13]. This could be an indication for intervention to encourage access of education for women.

The present study showed mothers, who worked as private employee, were about five times more knowledgeable about danger signs of pregnancy than housewives. The finding of this study is similar with a study conducted in Goba district of Ethiopia where mothers, who were government employees, were about four times more knowledgeable of danger signs compared to housewives [12]. This could be explained by the fact that, women who have their own source of income have better access to health related information.

Frequency of Antenatal care (ANC) visit was significantly associated with respondents’ knowledge about obstetric danger signs of pregnancy. Mothers having four ANC follow-ups were more knowledgeable about pregnancy danger signs by 91% than mothers having only one visit. Similarly, mothers who had four ANC follow-ups were 47% more likely to know danger signs of pregnancy than their mothers having only one ANC visit. This finding is consistent with a study done in Tanzania [11]. Similar studies conducted in Nigeria and different parts of Ethiopia showed that ANC visit was predictor of knowledge about danger signs during pregnancy, delivery and postpartum period [12, 19, 20]. This implies that stakeholders need to promote ANC follow-up including frequency of visits according to the standard.

In this study, women who gave birth to their last child in health institutions were about two times more likely to be knowledgeable about danger signs during delivery than women who gave birth at home. This finding is similar to a study conducted among rural Tanzanian women [11]. Another study conducted in East Gojjam zone and Tsegedie district of Ethiopia showed mothers who gave birth at health facilities were more knowledgeable about delivery danger signs [13, 14]. This could be an indication for promotion of institutional delivery to boost knowledge of mothers about danger signs in the study area.

As strength, this study employed a community-based approach when selecting the study participants. Additionally, recall bias was minimized by selecting mothers who gave birth recently (within 12 months). However, we cannot indicate the direction of causation to the associative relationships because of the nature of the study design.

## Conclusion and recommendations

Knowledge of mothers about obstetric danger signs was low in the study area. The most frequently mentioned obstetric danger sign during pregnancy, delivery, and postpartum period was vaginal bleeding. Maternal educational status, mothers’ occupation, number of ANC visit and institutional delivery were factors significantly associated with knowledge of obstetric danger signs. Empowering women, improving the quality of health information about danger signs during ANC follow up, and promoting institutional delivery are the recommended interventions.

## Abbreviations

ANC: Antenatal care
AOR: Adjusted odds ratio
CI: Confidence interval
COR: Crude odds ratio
EDHS: Ethiopian demographic and health survey
MMR: Maternal mortality ratio
OR: Odds ratio
SD: Standard deviation
SDG: Sustainable development goal
SPSS: Statistical package for social science
UN: United Nations’
